# Efficacy of PPV23 in Preventing Pneumococcal Pneumonia in Adults at Increased Risk – A Systematic Review and Meta-Analysis

**DOI:** 10.1371/journal.pone.0146338

**Published:** 2016-01-13

**Authors:** Julia Schiffner-Rohe, Annika Witt, Jana Hemmerling, Christof von Eiff, Friedrich-Wilhelm Leverkus

**Affiliations:** 1 Pfizer Deutschland GmbH, Linkstr. 10, 10785, Berlin, Germany; 2 AMS-Advanced Medical Services GmbH, Rosa-Bavarese-Str.5, 80639, Munich, Germany; 3 Pfizer Pharma GmbH, Linkstr. 10, 10785, Berlin, Germany; University of Dundee, UNITED KINGDOM

## Abstract

**Background:**

Pneumococcal community-acquired pneumonia (pCAP) is the most frequent form of pneumonia. The elderly and adults with underlying diseases are at an increased risk of developing pCAP. The 23-valent pneumococcal polysaccharide vaccine (PPV23) was licensed over 30 years ago and is recommended as the standard intervention in many countries across the globe, although its efficacy continues to be debated. We performed a meta-analysis of randomized controlled trials (RCTs) to investigate the effect of PPV23 for preventing pCAP in adults ≥60 years of age.

**Methods:**

An existing Cochrane Review was updated to Oct 2014 using a systematic literature search to select appropriate RCTs. DerSimonian and Laird random-effects meta-analyses were performed and odd ratios (OR) with 95%-confidence intervals (CI) and p-values were calculated for the descriptive analyses. Reasons for heterogeneity were explored by subgroup analyses.

**Results:**

Meta-analysis of PPV23 efficacy included four studies. Three of them did not demonstrate efficacy for PPV23. The body of evidence indicated statistically significant heterogeneity (*I*^*2*^ = 78%, p = 0.004) that could be explained by subgroup analysis by “study setting”. Further effect modifiers for pCAP were “continent of trial” (p<0.01), and “method of pneumococcal diagnostics” (p = 0.001). Subgroup analyses revealed that the only study showing efficacy for PPV23 was an outlier. Overall, the validity of the meta-analytic PPV23 efficacy assessment was confirmed by the meta-analysis of all-cause CAP including six studies.

**Discussion:**

Inconsistencies in PPV23 treatment effects to prevent pCAP could solely be explained by one outlier study that was performed in nursing homes in Japan. The effect modifier “method of pneumococcal diagnostics” should be interpreted carefully, since methodological weaknesses are not restricted to one special method only, which would justify the exclusion of certain studies. Overall, we conclude from our meta-analysis that to date there is no proof that PPV23 can prevent pCAP in a general, community-dwelling elderly population.

## Introduction

Pneumonia is a respiratory infection of the lungs which is caused mainly by viruses, fungi or bacteria. A distinction is made between community-acquired pneumonia (CAP), which is defined as pneumonia acquired outside a hospital, and hospital-acquired pneumonia (HAP). Pneumococci (*Streptococcus pneumoniae*, *SP*) are by far the most frequent pathogens recovered from patients with CAP (pCAP) and cause 12–85% of all CAP cases according to a recent systematic review which included 33 studies [1–3]. PCAP affects people of all ages, but children younger than five years of age, people with certain underlying medical conditions and the elderly (adults older than 60 years of age) are at higher risk and are more likely to experience a more severe course of the disease as well as complications, including death [4]. Comorbidities especially pulmonary disease may affect the risk of developing pneumococcal diseases and the efficacy of a pneumococcal vaccination [5–7].

Due to the high burden of disease, prevention of pneumococcal pneumonia, especially the non-bacteremic pCAP as the most frequent form, is one of the most important public health goals today.

There are two types of vaccines against pneumococci available, i.e. pneumococcal polysaccharide vaccine (PPV) and pneumococcal conjugate vaccine (PCV), in which the bacterial polysaccharide is covalently conjugated to an immunogenic carrier protein. PPV23 (Pneumovax^®^) contains 23 antigens of a total of 93 pneumococcal capsular serotypes, whereas the conjugate vaccines contain 7 (PCV7, Prevenar^®^), 10 (PCV10, Synflorix^®^) or 13 (PCV13, Prevenar 13^®^) polysaccharide antigens. Only PPV23 and PCV13 are currently licensed for adults.

PPV23 was licensed over 30 years ago and is recommended as standard intervention for the elderly (≥60 years of age) and for adults with underlying diseases in many countries around the globe [8] including the Standing Committee on Vaccination (STIKO) at the Robert Koch Institute (RKI) in Germany [9], although its efficacy continues to be debated [5, 10–12]. The impact of this vaccine on the epidemiology of pneumococcal infections in general or on noninvasive pCAP in particular has not been definitively ascertained anywhere in the world. This is particularly noteworthy for the two countries with a long history of PPV23 vaccination, the United States and United Kingdom (England and Wales) [13–15]. A recent meta-analysis by Moberley *et al*. concluded that PPVs are not effective in preventing death in adults [12]. Meta-analysis by Moberley *et al*. [12] used a diverse study pool that included different types of PPVs (PPV6, PPV12, PPV14 and PPV23) and the results indicated a substantial heterogeneity of the common effect estimates (*I*^*2*^ = 75% and 85%) for pneumonia and pneumococcal pneumonia, respectively. Furthermore the PPV23 efficacy results were not evaluated by age of participants. Therefore, uncertainties remain regarding the efficacy of PPV23, especially in elderly people [5].

In contrast, PCV13 has been shown to be effective in preventing pCAP in the elderly [16]. To date, no head-to-head studies have been published which compared PCV13 and PPV23 directly. To compare these two vaccines, an indirect comparison is required and therefore a new meta-analysis of efficacy assessments of the vaccines is needed. In this systematic review we reselected and updated the existing evidence of the Cochrane Review by Moberley *et al*. [12] to perform a meta-analysis of available randomized controlled trials (RCTs) investigating the efficacy of PPV23 on pCAP in adults at increased risk (elderly or adults with underlying diseases). In addition, reasons for uncertainties regarding the efficacy of PPV23 in the prevention of pCAP were explored by testing for heterogeneity and conducting subgroup analyses.

## Methods

### Literature search

We performed a systematic literature search to identify studies that investigated the efficacy of PPV23-intervention to prevent the outcome of CAP in a population of elderly (aged 60 and older) and adults with underlying diseases. At the time of the literature search the outcome pCAP was not specifically considered as a selection criterion.

RCTs that had been identified in the Cochrane Review by Moberley had only addressed the question of PPV efficacy in adults [12] and were therefore re-selected according to our selection criteria (Table 1) in order to identify studies that analysed explicitly the PPV23 efficacy to prevent pCAP. In addition to the existing evidence published by Moberley, the systematic literature search was updated to identify studies that were published thereafter.

**Table 1:** Selection criteria according to the STIKO.

	Inclusion criteria	Exclusion criteria
**Population**	Subjects ≥60 years	Studies including only HIV-positive subjects
	Subjects ≥18 and <60 years with high-risk factors:	
	Congenital or acquired immunodeficiencies or immunosuppression (except HIV-infection), e.g.:	
	T-cell deficiency or disordered t-cell function	
	B-cell- or antibody-deficiency (e.g. hypogammaglobulinaemia)	
	Deficiency or malfunction of myeloid cells (e.g. neutropenia, chronic granulomatous disease, leucocyte adhesion deficiencies, signal transduction deficiencies)	
	Complement or properdin deficiency	
	functional hyposplenism (e.g. during sickle cell anemia), splenectomy or anatomic asplenia	
	Neoplastic diseases	
	After bone marrow transplantation	
	Immunosuppressive therapy (e.g. due to organ transplantation or autoimmune disease)	
	Chronic diseases, e.g.:	
	Chronic diseases of the heart, the respiratory organs (e.g. asthma, emphysema, COPD), the liver or the kidney	
	Metabolic diseases, e.g. diabetes mellitus	
	Neurological diseases, e.g. cerebral palsy or seizure disorders	
	Anatomic and foreign body-associated risks for pneumococcal meningitis, e.g.	
	CSF fistula	
	Cochlear implant	
**Intervention**	Vaccination with PPV23	Vaccination with lower-valent Pneumococcal Polysaccharide Vaccines
		Vaccination with conjugate vaccines
**Control group**	Placebo or no intervention. Other non-pneumococcal vaccines administered as background vaccine to subjects in all study groups were permitted	
**Endpoints**	CAP	Studies investigating immunogenicity, safety etc. only
		
**Study type**	RCT	
**Type of publication**	Full publication or report in German, English or French language available, complying with CONSORT statement and allowing an assessment of the study results	No full publication or report available

Abbreviations: PPV23–23-valent pneumococcal polysaccharide vaccine; CAP—Community-Acquired Pneumonia; RCT–randomized controlled trial

In detail, the literature search by Moberley *et al*. 2013 was conducted in the databases CENTRAL 2012, Issue 6, MEDLINE (January 1966 to June Week 2, 2012) and EMBASE (1974 to June 2012). For the literature search update (2012–2014) the databases Cochrane Central Register of Controlled Trials (August 2014), MEDLINE (1946 to October 2014) and EMBASE (1947 to October 2014) were used (supporting information (S1–S3 Tables). The search was restricted to English, French or German as publication language. Because PPV23 vaccination is recommended for the elderly (≥60 years of age) and for adults with underlying diseases by the German STIKO [9] (and by many other recommending bodies in Europe and worldwide), we applied selection criteria which were based on the STIKO recommendations to the body of literature evidence in order to ensure transferability of the results from this meta-analysis to the context of the German health care system (Table 1).

### Study selection

Inclusion and exclusion criteria were applied to the body of evidence. Briefly, two independent persons (JH, MJ) re-selected full-text articles that were identified by Moberley [12] and selected studies identified from the literature search update for the years 2012–2014 according to their titles and abstracts with a subsequent selection of full-text publications from included studies (Table 1). Inconsistencies, if applicable, were clarified by a third person.

### Data extraction

Data sources for our meta-analysis were the selected publications on PPV23 vaccination. Studies were evaluated for eligibility for inclusion in the meta-analysis and data were extracted by two independent persons (JSR, AW) using appropriate forms, e.g. the data extraction form by the Cochrane working group (http://www.cochrane.org/sites/default/files/uploads/forums/u389/ERC%20data%20collection%20form%20for%20intervention%20reviews%20for%20RCTs%20only.doc). Inconsistencies were clarified and documented in clarification minutes, if applicable.

### Definition of endpoints for the review

#### All-cause community-acquired pneumonia (CAP)

For this systematic review, CAP was defined as pneumonia acquired in the community and diagnosed by the presence of clinical symptoms (fever, cough, dyspnoea or sputum) as well as a positive result of infiltrates on chest radiographs regardless of its cause. Cases of pneumonia without differentiation of acquisition mode were considered as “community-acquired pneumonia”, unless they were explicitly considered as HAP. Potential effects of nursing home settings (nursing home-acquired pneumonia, NHAP) on this assumption were further assessed in a subgroup analysis.

#### Pneumococcal community-acquired pneumonia (pCAP)

We defined pCAP as CAP with isolation of *SP* from sputum, bronchoaspirate, pleural fluid, blood, or cerebrospinal fluid OR a positive pneumococcal antigen test result in urine OR a positive pneumococcal antibody test result.

### Risk of bias assessment

Risk of bias in the included studies was evaluated for each study by assessing “random sequence generation” (selection bias), “allocation concealment” (selection bias), and “blinding of participants and personnel” (performance bias) (Table 2). RCTs were considered to provide the most valid results. However, in this review, we also allowed for inclusion of “pseudo-RCTs” where allocation to treatment group was not performed in a computer-generated random sequence, but still followed the principles of randomization. Further, we also allowed for open study designs where participants and personnel were aware of the treatment arm. We considered the risk of detection bias as low, because the diagnosis of pCAP is based on a concert of objective parameters such as clinical symptoms (fever, cough, dyspnea or sputum), a positive result of infiltrates on chest radiographs and the detection of *SP*. However, consequences of potential risk of bias were assessed by subgroup analyses.

**Table 2:** Risk of bias assessment of the identified clinical trials.

Study	Random sequence generation (selection bias)	Allocation concealment (selection bias)	Blinding of participants and personnel (performance bias) All outcome	Blinding of outcome assessment (detection bias) CAP / pCAP	Incomplete outcome data (attrition bias)	Selective outcome reporting (reporting bias)
Alfageme *et al*. 2006	Low risk	Unclear risk	High risk	Low risk	Low risk	Unclear risk
Oertqvist *et al*. 1998	Low risk	Low risk	Low risk	Low risk	Low risk	Unclear risk
Maruyama *et al*. 2010	Low risk	Low risk	Low risk	Low risk	Low risk	Unclear risk
Kawakami *et al*. 2010	Unclear risk	Low risk	High risk	Low risk	Low risk	Unclear risk
Furumoto *et al*. 2008	Low risk	Low risk	High risk	Low risk	Low risk	Unclear risk
Honkanen *et al*. 1999	Unclear risk	Unclear risk	High risk	Low risk	Low risk	Unclear risk

None of the publications reported on a potential bias for each assessed outcome. However, endpoints were not precisely defined in the publications and definitions of endpoints turned out to be diverse among selected clinical trials. To reduce the risk of bias for pCAP and CAP outcomes, we used medical expertise (DD) to link clinical endpoint definitions of the selected studies with our pCAP and CAP definitions (S4 Table).

Risk of bias across studies was explored by assessing degree of heterogeneity (see subgroup analysis for details).

### Statistical methods

#### Primary analysis

We used the DerSimonian and Laird random-effects method for meta-analyses (using the Cochrane Review Manager Version 5.3.) to evaluate possible heterogeneity of studies. In case of zero-cell counts, a fixed value of 0.5 was added to all cells of the respective study results tables [17]. We quantified between-trial heterogeneity using I-squared statistics and calculated standard Chi-square-tests of heterogeneity. Due to the lack of power of the standard test for heterogeneity, we interpreted p-values <0.2 or I-squared >50% as indicator for underlying heterogeneity [18]. Common effect sizes derived from the meta-analysis with a heterogeneous body of evidence were not considered as valid and therefore will not be presented. Analyses were based on the number of individuals who experienced the event of interest (pCAP or CAP), where applicable. Otherwise, number of episodes were used for analysis. We calculated odds ratios (OR) with 95%-confidence intervals (CI) and descriptive p-values. Where applicable, we presented results from individual trials and the common effect estimate in a forest plot. Squares indicate individual study odds ratios together with its 95% CI indicated as bars. Odds ratios below 1.0 correspond to an effect favouring PPV23 vaccination. The size of the square indicates the weight of the individual study in the meta-analysis. Estimates for the common odds ratio together with the 95% CI, as derived from the meta-analysis, are presented as diamonds.

#### Subgroup analysis

Reasons for heterogeneity were explored based on clinical considerations by assessing pre-defined subgroup criteria as potential effect modifiers. Effect modifiers were assessed using interaction tests. According to the rules for interaction tests as defined by the German Institute for Quality and Efficiency in Health Care (IQWiG) [18] we performed meta-analyses on *a priori* defined subgroups and tested subgroup differences (interpretation as interaction test–effect modifier). When the interaction test indicated effect modification (p-values below 0.2 give a hint of effect modification, p-values below 0.05 give a proof of effect modification), no common effect estimate was calculated and only subgroup effect estimates are presented. For both, pCAP and CAP outcomes, we *a priori* defined the following potential effect modifiers as being of interest: “study setting (community/nursing home)”, “continent of trial”, “age”, “trial duration”, “co-vaccination with influenza vaccine (IV) (yes/no)” and “high/low income countries”. The feasibility of subgroup analyses depended on the availability of the respective results in the publications and was discussed separately. As a consequence of identified studies we *a posteriori* defined additional subgroups for “trial quality (blinding and concealment of allocation)” and “pneumococcal diagnostics (Binax /PLY/none)”.

Unless specifically stated, all methods were defined *a priori* in a review protocol (data on file).

## Results

### Literature search and characteristics of included studies

**Fig 1:**
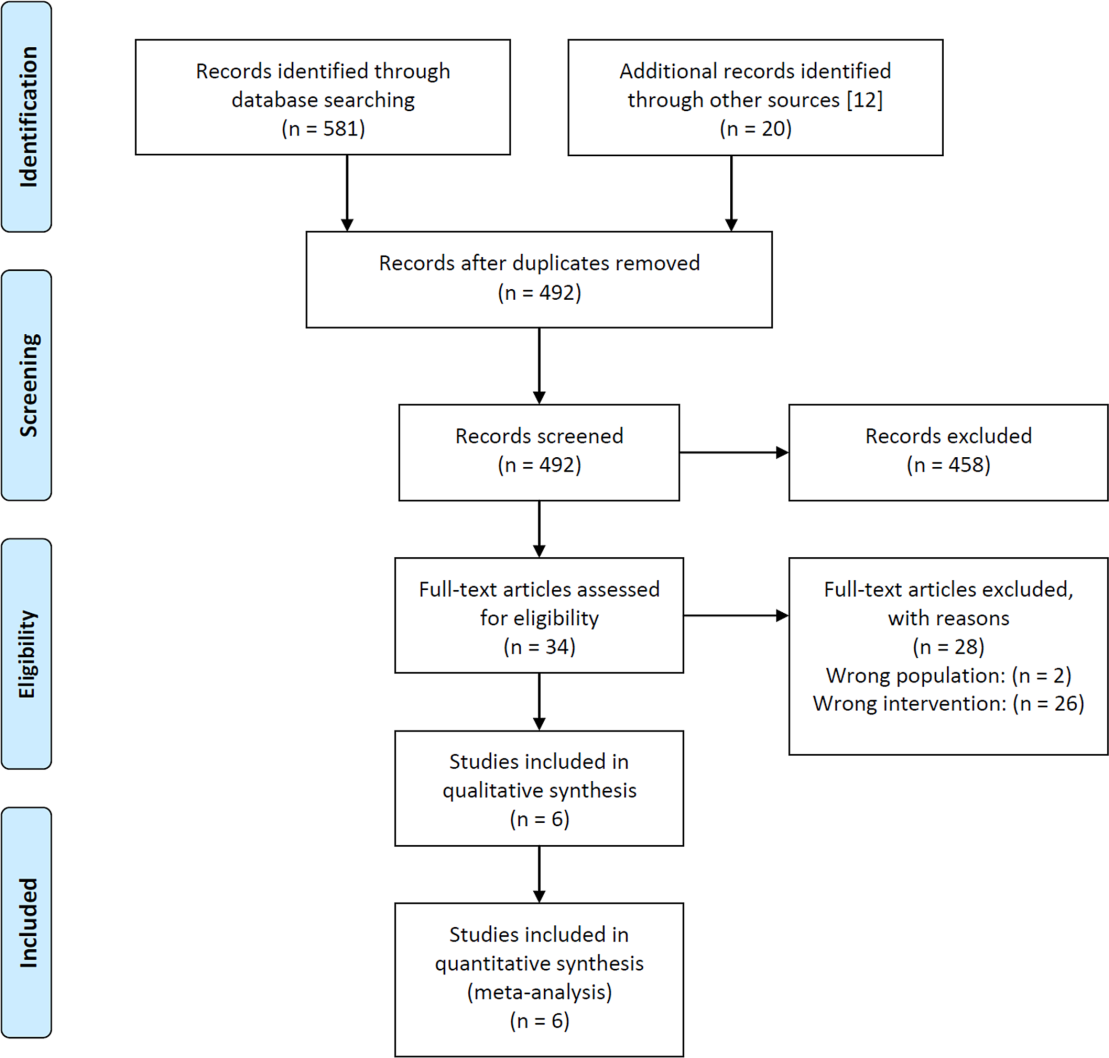
Integrative flowchart of relevant full-texts identified by literature search used in Moberley et al. 2013 and by literature search update from 2012–2014.

We screened 581 abstracts and 14 full-text articles, identified by the literature search update (2012–2014) according to the selection criteria on the basis of title and abstract. Additionally we reviewed 20 full-text articles from Moberley *et al*. 2013 (Fig 1).

The identified RCTs investigated the efficacy of PPV23 in the prevention of CAP, especially pCAP, in subjects ≥60 years of age. No study was identified that reported on PPV23 efficacy to prevent pCAP or CAP in adults, 18–60 years of age, with underlying diseases. Studies that did not match the selection criteria were excluded from the analysis, mostly due to wrong intervention (S5 Table, S6 Table). As a consequence, six RCTs (Alfageme *et al*. [19], Furumoto *et al*. [20], Kawakami *et al*. [21], Maruyama *et al*. [22], Honkanen *et al*. [23], Oertqvist *et al*. [24]) involving 30,171 subjects in total have been considered in this meta-analysis (Table 3). Our selected studies differed in several factors (Table 3), such as study setting, continent of trial and pneumococcal diagnostics. Baseline data are depicted in Table 4.

**Table 3:** Study characteristics of the identified clinical trials.

Identified study	Study type and total number of included participants^#^	Study setting	Underlying comorbidities	IV	Continent of trial	Follow-Up period	Diagnosis of CAP	Pneumococcal diagnostics
Alfageme *et al*. 2006	RCT	Community	COPD	Yes	Europe	up to 3 years	Clinical symptoms	Isolation of *SP* from the sputum (for an adequate sample), bronchoaspirate, blood, pleural fluid, or cerebrospinal fluid
	596 participants					PPV23: 980.0 days (≈2.68 years)	Radiography	
						no vaccination: 977.8 days (≈2.68 years)	Radiographic follow-up	
Furumoto *et al*. 2008	RCT	Community	CLD	Yes	Asia	2 years (no mean follow-up duration was reported)	Clinical symptoms (plus increased white blood cell counts or serum CRP)	None
	162 participants						Radiography	
							Radiographic follow-up	
Kawakami *et al*. 2010	RCT	Community	Chronic heart disease	Yes	Asia	2 years (no mean follow-up duration was reported)	Clinical symptoms	None
	778 participants		Hypertension				Radiography	
			CLD				Computed tomography	
			Chronic renal diseases					
			Prior episode of pneumonia					
			Difficulty of walking					
Maruyama *et al*. 2010	RCT	Nursing Home	Cerebrovascular disease	Yes	Asia	at least 26 months	Clinical symptoms	Cultures from blood, pleural fluid, or sputum (107 colony forming units per milliliter in a purulent sample) or pneumococcal antigen test (BinaxNOW^®^ in urine samples)
	1006 participants		Chronic pulmonary disease			PPV23: 2.27 years	Radiography	
			Malignancy			Placebo: 2.28 years	Radiographic follow-up	
			Psychological disorder				Computed tomography (approx.70% of subjects)	
			Diabetes mellitus					
			Congestive heart failure					
			Other heart disease					
			Chronic liver disease					
			Chronic renal disease					
			Gastrostomy					
			Gastrectomy					
			Post-surgical					
			Bone fracture					
			Hypertension					
			Hyperlipidaemia					
			Other					
Oertqvist *et al*. 1998	RCT	Community	Smoker	Not reported	Europe	Reported as exposure time	Clinical symptoms	Blood-cultures, quantitative sputum cultures from purulent samples, if possible PLY-antibody test (EIA) in serum samples
	691 participants		Alcoholic			PPV23: 2.3 years	Radiography	
			Chronic pulmonary disease			Placebo: 2.5 years	Radiographic follow-up	
			Heart failure					
			Other heart disease					
			Chronic liver disease					
			Diabetic					
			“Other” chronic diseases					
			Previously healthy					
			Previously healthy, non-smoker, non-alcoholic					
Honkanen *et al*. 1999	(Pseudo-) randomized cohort study	Community	Hypertension	Yes	Europe	3 years (cohort I)	Clinical symptoms	PLY-antibody test (EIA) in serum samples
			Congestive heart disease			2 years (cohort II)	Radiography	
	26,925 participants		Coronary heart disease				Radiographic follow-up	
			Diabetes mellitus					
			Asthma or COPD					
			Renal disease					
			Rheumatoid arthritis					
			Pernicious anaemia					
			Malignancy					

#: According to the final analysis-population of the respective study

Abbreviations: CAP: Community-Acquired Pneumonia; CLD: Chronic Lung Diseases; COPD: Chronic Obstructive Pulmonary Disease; CRP: C-reactive Peptide EIA: Enzyme-Linked Immunosorbent Assay; IV: Influenza Vaccination; PLY: Pneumolysin; PPV23: 23-valent Pneumococcal Vaccine; RCT: Randomised Controlled Trial; *SP*: *Streptococcus pneumoniae*

**Table 4:** Baseline characteristics of subjects^#^ included in the identified clinical trials.

Study	Intervention	Comparator
Alfageme *et al*. 2006	PPV23 (N = 298)	No vaccination (N = 298)
	Mean age (range): 69 (62–73) years	Mean age (range): 68 (61–73) years
	<65 years: n = 91 (31%)	<65 years: n = 116 (39%)
	≥65 years: n = 207 (69%)	≥65 years: n = 182 (61%)
	Male: 96.6%	Male: 93.3%
Oertqvist *et al*. 1998	PPV23 (N = 339)	Placebo (N = 352)
	Mean age (SD): 69.4 (±9.2) years	Mean age (SD): 69.1 (±9.0) years
	≤65 years: n = 102 (30%)	≤65 years: n = 126 (36%)
	>65 years: n = 237 (70%)	>65 years: n = 226 (64%)
	Male: 47.5%	Male: 48.0%
Maruyama *et al*. 2010	PPV23 (N = 502)	Placebo (N = 504)
	Mean age (SD): 84.7 (±7.7) years	Mean age (SD): 84.8 (±7.6) years
	<65 years: n = 9 (2%)	≤65 years: n = 11 (2%)
	≥65 years: n = 493 (98%)	>65 years: n = 493 (98%)
	Male: 22.1%	Male: 22.1%
Kawakami *et al*. 2010	PPV23 (N = 391)	No vaccination (N = 387)
	Mean age (SD): 78.5 (±7.3) years	Mean age (SD): 77.7 (±7.2) years
	<65 years: n = 0 (0%)	<65 years: n = 0 (0%)
	≥65 years: n = 391 (100%)	≥65 years: n = 387 (100%)
	Male: 38.1%	Male: 32.3%
Furumoto *et al*. 2008	PPV23+IV (N = 87)	IV (N = 80)
	Mean age (SD): 67.8 (±9.5) years	Mean age (SD): 70.1 (±7.4) years
	<65 years: not reported	<65 years: not reported
	≥65 years: not reported	≥65 years: not reported
	Male: 69.0%	Male: 57.5%
Honkanen *et al*. 1999	PPV23+IV	IV
	N = 4,902 (cohort I), N = 9,078 (cohort II)	N = 4,973 (cohort I), N = 7,972 (cohort II)
	Mean age (SD):	Mean age (SD):
	74.1 (±6.8) years (cohort I)	73.9 (±7.0) years (cohort I)
	72.8 (±6.5) years (cohort II)	73.6 (±6.5) years (cohort II)
	<65 years: n = 0 (0%)	<65 years: n = 0 (0%)
	≥65 years: n = 13,980 (100%)	≥65 years: n = 12,945 (100%)
	Male: 38.6% (cohort I), 38.2% (cohort II)	Male: 38.6% (cohort I), 37.4% (cohort II)

# According to the final analysis-population of the respective study

Abbreviations: IV:Influenza Vaccine; N: Number of Individuals per Population, n: Number of Individuals per Subpopulation; PPV23: 23-valent Pneumococcal Vaccine; SD: Standard Deviation

All trials reported data on CAP; pCAP endpoints were evaluated in four out of six studies: Alfageme *et al*. [19], Maruyama *et al*. [22], Honkanen *et al*. [23] and Oertqvist *et al*. [24]. In contrast to the other studies, Honkanen *et al*. [23] reported number of episodes instead of number of patients with CAP or pCAP.

A correct diagnosis of pCAP remains challenging, but is crucial for the assessment of the efficacy of the vaccine to prevent pCAP. The reliability of microbiological diagnosis depends on the technique that is used and on how the samples are selected [25]. Culture-based techniques using blood or “qualified sputum” are still the gold standard due to their high specificity, although sensitivities of these methods are limited. In order to improve the sensitivity of the pneumococcal CAP diagnosis, alternative or additional detection methods were developed. All of the four studies selected for the meta-analysis of PPV23 efficacy in pCAP [19, 22–24] used established microbiological confirmatory tests to secure pCAP diagnosis from blood (in case of invasive pCAP) or (qualified) sputum samples. Three studies (Honkanen *et al*. [23], Maruyama *et al*. [22] and Oertqvist *et al*. [24]) additionally used tests to detect pneumococci from serum or urine samples. One study (Maruyama *et al*. [22]) used the commercially available urinary BinaxNOW^®^ assay that detects *SP*-specific C-polysaccharides, while Oertqvist *et al*. [24] and Honkanen *et al*. [23] used an enzyme immunoassay (EIA)-based method to detect IgG antibodies specific to pneumolysin (PLY) (anti-PLY IgGs EIA) recovered from precipitated circulating immune complexes (CICs) from serum specimen. Alfageme *et al*. did not use any further test to detect pneumococci [19]. Therefore we additionally considered the pneumococcal diagnostics as a potential effect modifier in a subsequent subgroup analysis (see section Analysis of *a posteriori* defined subgroups).

### Quality of included studies

In general, the comparability between trials is limited due to different study settings, populations, age groups and continents (Table 3). This issue will be discussed as a limitation for the subsequent meta-analysis (see discussion).

Definitions of pCAP turned out to be diverse among selected clinical trials. We therefore linked clinical endpoint definitions of the selected studies with our pCAP definition (S4 Table).

Risk of bias assessment was performed on a study basis (Table 2).

### Meta-analytic efficacy assessment of PPV23 to prevent pCAP

Meta-analysis of pCAP, the primary outcome for the assessment of PPV23 efficacy, was based on Alfageme *et al*. [19], Maruyama *et al*. [22], Honkanen *et al*. [23] and Oertqvist *et al*. [24] (Table 5). Meta-analysis revealed a statistically significant heterogeneity (*I*^*2*^ = 78%, p = 0.004), indicating that study results differed to an extent that were not explainable by chance and therefore comparability between studies was limited. As a consequence, the meta-analysed effect estimate was evaluated as not valid. A possible systematic effect had to be taken into account and we therefore searched for potential reasons by subgroup analysis.

**Table 5:** Primary analysis of the number^#^ of patients experiencing a pCAP.

Comparison	PPV23		Comparator		Treatment effect
Study	N	n (%)	N	n (%)	OR [95%-CI]; p-value
Oertqvist *et al*. 1998	339	19 (5.6)	352	16 (4.5)	1.25 [0.63;2.47]; 0.53
Honkanen *et al*. 1999^a^	13,980	52 (0.4)	12,945	40 (0.3)	1.20 [0.80;1.82]; 0.38
Alfageme *et al*. 2006	298	0 (0)	298	5 (1.7)	0.09 [0.00;1.62]; 0.10
Maruyama *et al*. 2010	502	14 (2.8)	504	37 (7.3)	0.36 [0.19;0.68]; 0.002

#: According to the final analysis-population of the respective study

a: In contrast to the other studies, Honkanen *et al*. reported number of episodes instead of number of patients with pCAP.

Abbreviations: CI: Confidence interval; N: Number of Individuals per Population, n: Number of Individuals per Subpopulation; OR: Odds Ratio; PPV23: 23-valent pneumococcal polysaccharide vaccine

When interpreting the results of analysis, it is important to take into consideration that the sample size of Alfageme *et al*. was very small in relation to the rate of observed pCAP (0 versus 5 cases) and consequently the estimates of treatment effect in this individual study are prone to high variability [19].

### Subgroup analysis of pCAP

To further analyse the reasons for uncertainties regarding the efficacy of PPV23 in the prevention of pCAP, subgroups were explored. We considered the following *a priori* defined potential effect modifiers: “study setting (community/ nursing home)”, “continent of trial (Europe/Asia)” and “age (≥65/<65)” for pCAP. Analyses of all other *a priori* defined subgroups were not feasible as either published data were not precise enough (“co-vaccination with IV” and “trial duration”) or all studies belonged to the same subgroup, e. g. “income of country”.

The identified studies used different assays to detect *SP* which might have influenced the number of diagnosed pCAP cases due to different spectra of test sensitivity and specificity. Therefore, “pneumococcal diagnostics (Binax/PLY/none)” was targeted as *a posteriori* defined potential effect modifier below. Since study qualities were different especially due to “allocation concealment (computer generated random numbers/other)” and “blinding (double blind/open)” further subgroup analyses according to a potential risk of selection bias were performed.

#### Analysis of *a priori* defined subgroups

**Fig 2:**
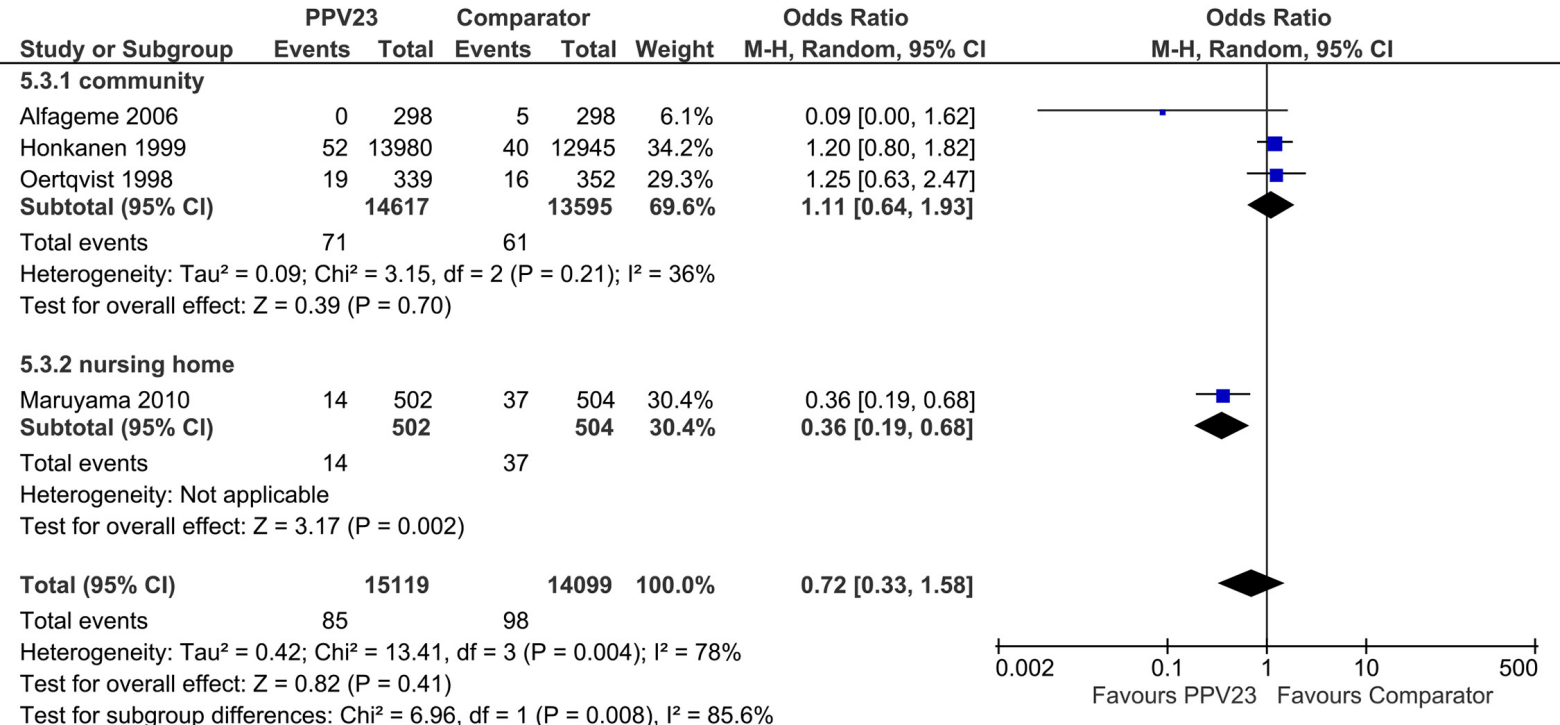
Forest Plot of PPV23 efficacy to prevent pCAP stratified by study setting.

The subgroup “study setting” (community/nursing home) revealed significant subgroup differences (p = 0.008) (Fig 2). Heterogeneity of the pCAP outcome from the primary analysis could be explained (*I*^*2*^ = 36%, p = 0.21). However, no significant treatment effect of PPV23 was observed for the community setting (OR [95%-CI]: 1.11 [0.64; 1.93]; heterogeneity: p = 0.70). This is different in nursing home patients (OR [95%-CI]: 0.36 [0.19; 0.68]). It has to be kept in mind however, that this finding is derived from a single trial (Maruyama *et al*. [22]) only.

Subgroup analysis by “continent of trial” (Europe/Asia) was identical with the analysis according to the study setting since Maruyama *et al*. [22], the only study conducted in a nursing home, was also the only study not conducted in Europe (Table 6).

**Table 6:** Key results of the meta-analysis.

Effect estimate	Number of trials	OR [95%-CI]	Heterogeneity *I*^*2*^(%); p-value	Subgroup differences *I*^*2*^(%); p-value
**pCAP**				
**Overall estimate**	4	n.e.*	78%; p = 0.004	
**Setting**	4			85.6%; p = 0.008
Community	3	1.11 [0.64;1.93]	36%; p = 0.21	
Nursing home	1	0.36 [0.19;0.68]	n.a.	
**Continent**	4			85.6%; p = 0.008
Europe	3	1.11 [0.64;1.93]	36%; p = 0.21	
Asia	1	0.36 [0.19;0.68]	n.a.	
**Age**	3			68.4%; p = 0.08
≥65	3	n.e.*	81%; p = 0.005	
<65	1	0.21 [0.06;0.76]	n.a.	
**Allocation concealment**	4			43.4%; p = 0.18
Computer generated random numbers	3	n.e.*	77%, p = 0.01	
Other	1	1.20 [0.80;1.82]	n.a.	
**Blinding**	4			0%; p = 0.83
Double blind	2	n.e.*	85%, p = 0.009	
Open	2	n.e.*	68%, p = 0.08	
**Pneumococcal diagnostic**	4			84.9%; p = 0.001
Binax	1	0.36 [0.19;0.68]	n.a.	
PLY	2	1.22 [0.85;1.73]	0%; p = 0.93	
None	1	0.09 [0.00;1.62]	n.a.	
**All- cause CAP**				
**Overall estimate**	6	n.e.*	63%; p = 0.02	
**Setting**	6			92.0%; p = 0.0004
Community	5	1.10 [0.93;1.30]	0%; p = 0.91	
Nursing home	1	0.55 [0.39;0.78]	n.a.	
**Continent**	6			63.3%; p = 0.10
Europe	3	1.13 [0.94;1.38]	0%; p = 0.80	
Asia	3	0.76 [0.49;1.17]	58%; p = 0.09	
		when excluding nursing home setting:	when excluding nursing home setting:	
		0.97 [0.67;1.42]	0%; p = 0.95	
**Age**				n.a. (no stratified data)
**Allocation concealment**	6			0%; p = 0.34
Computer generated random numbers	3	n.e.*	77%, p = 0.01	
Other	3	1.10 [0.90;1.35]	0%, p = 0.74	
**Blinding**	6			0%; p = 0.45
Double blind	2	n.e.*	88%, p = 0.004	
Open	4	1.08 [0.89;1.31]	0%, p = 0.85	

Abbreviations: n.a.: not applicable

*n.e.: no valid estimate due to heterogeneity in underlying evidence; CI: Confidence Interval; PLY: pneumolysin; CAP: Community-Acquired Pneumonia, pCAP: Pneumococcal Community-Acquired Pneumonia

Subgroup analysis by “age” (≥65/<65 years of age, interaction test: p = 0.08; Table 6) showed strong heterogeneity within the subgroup “≥65 years” (*I*^*2*^ = 81.0%, p = 0.005) leading to an invalid effect estimate. A reason may be that the subgroup of participants “≥65 years” was comprised of pooled data from Alfageme *et al*. [19], Maruyama *et al*. [22] and Honkanen *et al*. [23]. It should particularly be noted, that the included data from Alfageme derived from a different age-stratified pCAP endpoint, which was defined as ‘presumptive CAP of pneumococcal or unknown aetiology’.

#### Analysis of *a posteriori* defined subgroups

In subgroup analysis, by “allocation concealment” (interaction test: p = 0.18) heterogeneity could not be solved by data stratification leading to invalid meta-analysed effect estimates (Table 6). It was shown that “blinding” is not an effect modifier (interaction test: p = 0.83).

In contrast, analysing data stratified by “pneumococcal diagnostics” (Binax/PLY/none) showed significant subgroup differences (interaction test: p = 0.001) (Table 6). Studies using pneumolysin antibody tests (“PLY”) did not demonstrate an effect of PPV23 to prevent pCAP (*I*^*2*^ = 0%, p = 0.93; OR: 1.22 [0.85; 1.73]) and are in line with the study not using any additional diagnostic tool (“none”). The subgroup of studies using BinaxNow^®^ assay (“Binax”) revealed a significant PPV23 treatment effect. However, these data were exclusively based on the study of Maruyama *et al*. which was the only study conducted in Asia and also belonged to the subgroup “nursing home” [22].

In summary, heterogeneity of the pCAP outcome from the primary analysis could be explained by the subgroup analyses according to the study setting. Further effect modifiers for pCAP were “continent of trial”, and “pneumococcal diagnostics”. In all cases, except for “age”, Maruyama *et al*. [22] was driving the subgroup effect, being: 1) the only study that was conducted in a nursing home, 2) the only non-European study and 3) the only study using BinaxNow^®^ assay as diagnostic tool. These findings point to the outlier characteristics of the study [22], which is supported by the fact, that all other studies, except the study by Maruyama *et al*.[22], showed no proof that PPV23 can prevent pCAP.

### Meta-analytic efficacy assessment of PPV23 to prevent all-cause CAP is consistent with the analyses of pCAP

To check the validity of the meta-analysis regarding pCAP we compared the results with the endpoint CAP (Table 7). CAP was reported in all six trials identified by literature search (Alfageme *et al*. [19], Furumoto *et al*. [20], Kawakami *et al*. [21], Maruyama *et al*. [22], Honkanen *et al*. [23], Oertqvist *et al*. [24]). Comparable to pCAP efficacy results to prevent CAP, showed a statistically significant heterogeneity (*I*^*2*^ = 63%, p = 0.02), indicating that study results differed to an extent that were not explainable by chance. Five of the six studies showed no treatment effect of PPV23 to prevent CAP. Only the study published by Maruyama *et al*. showed a significant effect estimate favouring PPV23 (OR [95%-CI] 0.55 [0.39; 0.78]) [22]. As a consequence of heterogeneity, the overall common effect estimate was evaluated as not valid.

**Table 7:** Primary analysis of the number^#^ of patients experiencing an all-cause CAP.

Comparison	PPV23		Comparator		Treatment effect
Study	N	n (%)	N	n (%)	OR [95%-CI]; p-value
Alfageme *et al*. 2006	298	33 (11.1)	298	34 (11.4)	0.97 [0.58;1.61]; 0.90
Furumoto *et al*. 2008	87	13 (14.9)	80	12 (15.0)	1.00 [0.43;2.33]; 0.99
Honkanen *et al*. 1999^a^	13,98	145 (1.0)	12,945	116 (0.9)	1.16 [0.91;1.48]; 0.24
Kawakami *et al*. 2010^a^	391	49 (12.5)	387	50 (12.9)	0.97 [0.63;1.47]; 0.87
Maruyama *et al*. 2010	502	63 (12.5)	504	104 (20.6)	0.55 [0.39;0.78]; 0.0006
Oertqvist *et al*. 1998	339	63 (18.6)	352	57 (16.2)	1.18 [0.80;1.75]; 0.41

#: According to the final analysis-population of the respective study

a: In contrast to the other studies, Honkanen *et al*. reported number of episodes instead of number of patients with CAP.

Abbreviations: CI: Confidence interval; N: Number of Individuals per Population, n: Number of Individuals per Subpopulation; OR: Odds Ratio; PPV23: 23-valent pneumococcal polysaccharide vaccine

### Subgroup analysis of CAP

As specified in the pCAP analysis we again searched by subgroup analyses for potential reasons that might explain the heterogeneity of primary CAP results. *A priori* defined potential effect modifiers were “study setting (community/ nursing home)” and “continent of trial”. A subgroup analysis by “age” was not possible due to a lack of age-stratified CAP data in the identified studies. *A posteriori* defined potential effect modifiers were “allocation concealment” and “blinding”. Since all-cause CAP does not rely on the diagnostic measures for pneumococcal participation, the *a posteriori* subgroup “pneumococcal diagnostics” was not relevant for this endpoint.

#### *A priori* defined subgroups

**Fig 3:**
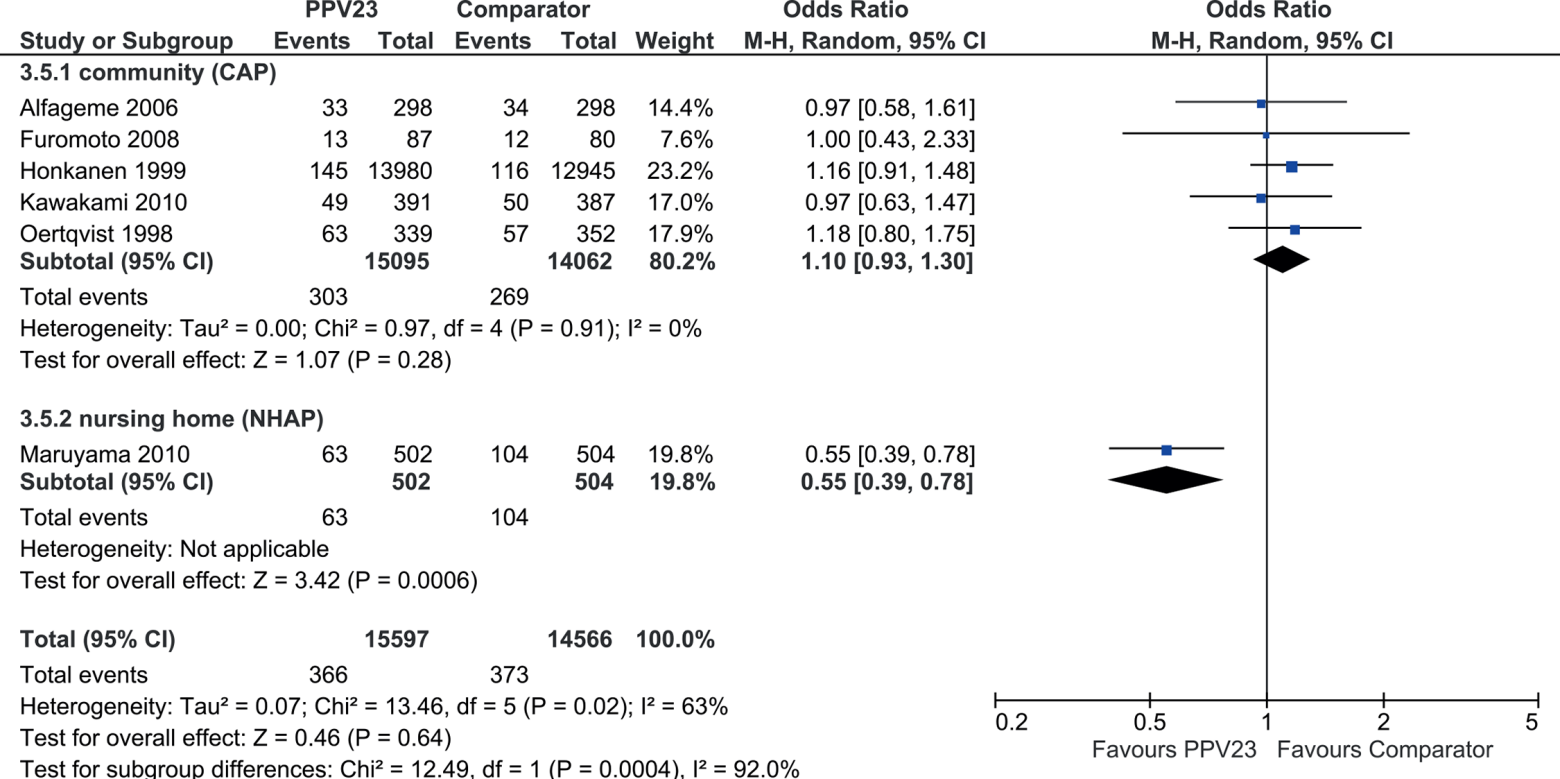
Forest Plot of PPV23 efficacy to prevent all-cause CAP by study setting.

Comparable to the pCAP analysis, study setting turned out to be a relevant effect modifier since there was a remarkable and significant subgroup difference (p = 0.0004), (Table 6). Stratification of CAP data by study setting explained the inter-study heterogeneity of the common effect estimate for CAP in “community” [*I*^*2*^ = 0%; p = 0.91] (Fig 3). However, and consistent with the pCAP analysis, meta-analysis of the subgroup “community” showed no treatment effect of PPV23 intervention to prevent all cause CAP. Only data from the subgroup “nursing home” indicate a significant treatment effect for PPV23. Since data from this subgroup were again based on a single study (Maruyama *et al*. [22]) meta-analysis was not possible.

**Fig 4:**
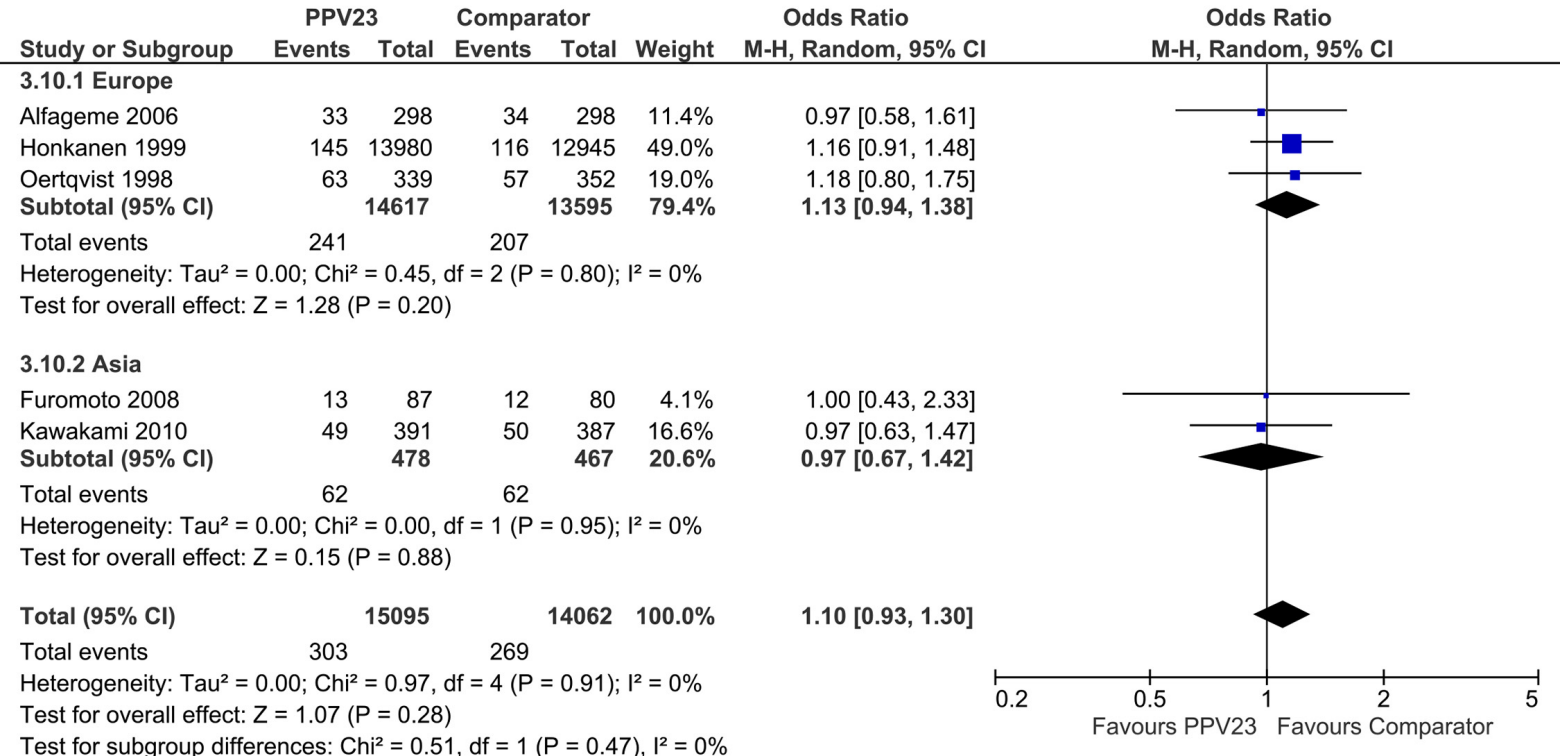
Forest Plot of PPV23 efficacy to prevent all-cause CAP by continent of trial (without study setting “Nursing home”).

For the CAP analysis, in contrast to the pCAP analysis, three studies (Maruyama *et al*. [22], Furumoto *et al*. [20] and Kawakami *et al*. [21]) were conducted in Asia. The subgroup analysis of “continent of trial” showed that this subgroup was an effect modifier, too (Table 6). Meta-analysis by subgroup revealed no treatment effect of PPV23 in European trials (“Europe”) [19, 23, 24], whereas the common effect estimate supported an effect of PPV23 in studies performed in Japan (“Asia”) [20–22] (OR [95%-CI] 0.76 [0.49;1.17]). However, data from the Asian subgroup remain heterogeneous (*I*^*2*^ = 58%, p = 0.09). We hypothesized that the study by Maruyama *et al*. [22] was an outlier study and therefore addressed this question in a subsequent sensitivity analysis where we excluded the study of Maruyama *et al*. [22] from the subgroup analysis (Fig 4), which solved the heterogeneity within the subgroup of Asian studies (“Asia”) (*I*^*2*^ = 0%, p = 0.95). This strengthens our hypothesis and again indicated that Maruyama *et al*. [22] was an outlier study.

#### *A posteriori* defined subgroups

Subgroup analyses of allocation concealment and blinding, revealed no subgroup differences (p = 0.34 and p = 0.45), respectively (Table 6).

In summary, the meta-analysed efficacy results of PPV23 to prevent pCAP are consistent with the meta-analysis of PPV23 effect estimates to prevent CAP (Table 6). Both, pCAP and CAP endpoints showed any significant treatment effect of PPV23 intervention in a community setting. “Study setting” and “continent of trial” were effect modifiers. Additionally, for the pCAP endpoint the method of “pneumococcal diagnostics” influenced the outcome. Consistently, in all subgroup analyses, the study by Maruyama *et al*. [22] was always in a distinct subgroup compared to the other studies, suggesting that heterogeneities in the meta-analyses were driven by this outlier characteristic.

## Discussion

Many countries including Germany [9] recommend PPV23 as a standard vaccination to all elderly ≥60 years of age or to adults with underlying diseases, even though its efficacy is questioned [5, 10–12]. The only currently licensed non-conjugated PPV is PPV23. In this context our meta-analysis was designed to estimate the efficacy of PPV23 in the prevention of pCAP, particularly in patients above 60 years of age and adults with underlying diseases. Furthermore, the validity of this meta-analysis was confirmed by comparing the results for pCAP with the endpoint CAP and reasons for uncertainties regarding the effectiveness of PPV23 in preventing pCAP were explored.

Overall, no study was identified that investigated the efficacy of PPV23 in preventing pneumonia in adults younger than 60 years (18–60 years of age) with underlying diseases. In total six studies (Alfageme *et al*. [19], Furumoto *et al*. [20], Kawakami *et al*. [21], Maruyama *et al*. [22], Honkanen *et al*. [23], Oertqvist *et al*. [24]) were identified investigating the efficacy of PPV23 in preventing pneumonia in the elderly (≥60 years). Four of the six studies [19, 22–24] reported pCAP endpoints and could be used for meta-analysis of pCAP, the endpoint of primary interest in this study. The results of the meta-analysis were heterogeneous showing that the comparability between studies was limited. To identify potential effect modifiers, *a priori* defined subgroups as well as *a posteriori* defined subgroups were explored. Significant effect modifiers for pCAP were “study setting”, “continent of trial” and “pneumococcal diagnostics”. The heterogeneous results of the pCAP outcome could solely be explained by one outlier study, Maruyama *et al*. [22]. In all subgroup analyses that explained the heterogeneity, Maruyama *et al*. [22] was: 1) the only study that was performed in a nursing home, 2) the only non-European study and 3) the only study using BinaxNow^®^ assay as diagnostic tool. The other studies in contrast were community-based and conducted in Europe. Subgroup analyses of CAP supported these findings.

In detail, a remarkable effect modifier in pCAP and CAP analysis was “study setting”. Heterogeneity of the pCAP/CAP outcomes from the primary analyses could be explained by subgroup analyses (nursing home versus community). However, within the subgroup “community” there was no PPV23 efficacy to prevent pCAP. In contrast, in a nursing home environment PPV23 was effective in the prevention of pCAP, but it need to be kept in mind that this finding is based on a single trial (Maruyama *et al*. [22]), which was evaluated as an outlier. This effect of the study setting on PPV23 efficacy might be due to the fact that microbial causes of CAP may differ between nursing homes compared to the community (e.g. by possible outbreaks with certain pneumococcal serotypes in a nursing home) which might result in a different efficacy of PPV23 intervention [26]. Considerations of a high infectious pressure in nursing homes due to distinct ways of infections (e.g. conditions of living together, by sharing of silverware, etc.) support this observation.

Another significant effect modifier for both, pCAP and CAP meta-analyses was “continent of trial”. Further investigations on the subgroup level, however, were only possible for CAP, since three of the six studies (Maruyama *et al*. [22], Furumoto *et al*. [20] and Kawakami *et al*. [21]) were conducted in Asia. Taking all three studies into consideration, remarkable inner-subgroup heterogeneity remained. Sensitivity analysis by exclusion of the study by Maruyama *et al*. [22] could solve inner-subgroup heterogeneity, again supporting the finding that this study has to be interpreted as an outlier. Nevertheless, results of “continent of trial” show that the impact of PPV23 vaccination differs among ethnicities.

Beside “study setting” and “continent of trial” we identified “pneumococcal diagnostics” for *SP* as a significant effect modifier for pCAP.Due to the limited sensitivity of established methods for diagnosing pneumonia and for microbiological confirmatory tests [25], additional (but different) methods to detect pneumococci were used in three (Honkanen *et al*. [23], Maruyama *et al*. [22], Oertqvist *et al*. [24]) of the four pCAP studies (Table 3). The assumed high specificity and sensitivity of the BinaxNOW^®^ assay has been questioned several times [27–29]. In contrast, the anti-PLY IgG EIA used by Oertqvist *et al*. [24] and Honkanen *et al*. [23] was validated and developed by the group of M. Leinonen [23, 24, 30, 31]. Two subsequent publications (Scott *et al*. [32] and Musher *et al*. [33]) have questioned the validity of the PLY-detection method by Jalonen *et al*. [30]. However, these publications have their own methodological weaknesses again questioning the reliability of any judgement on the PLY-detection method: A small validation trial on this assay by Scott *et al*. [32] concluded that “the sensitivities of the enzyme immuno assay (EIA) could exceed that of blood cultures only at levels of specificity that were insufficient for the performance of vaccine efficacy studies”. However, this study has several limitations (incl. no proper characterization of the “sick” control group, no evaluation of assay specificity in healthy subjects and high rate of HIV co-infected patients) making a judgement about limited specificity and poor to moderate sensitivity in different subgroups difficult. The authors of the Musher *et al*. study [33] stated that this method is not a reliable method for diagnosing pneumococcal pneumonia. However, this study itself was associated with major limitations since comparison of the methodological part of Jalonen *et al*. [30] and Musher *et al*. [33] reveals a significant deviation in the coating antigen used to capture anti-PLY IgGs. Jalonen *et al*. used purified PLY from a type 1 strain of *SP*) [30] and Musher *et al*. used pneumolysoid B, a recombinant protein toxoid derivative of PLY, which was expressed and purified from *Escherichia coli* [34]. To our best knowledge, a validation of pneumolysoid B as a replacement for the PLY antigen in the EIA to detect anti-PLY IgG has never been published. To date, the PLY/CIC-assay is a validated method for detection of pneumococcal involvement, although it cannot be fully excluded that this assay might also slightly overestimate the rate of pCAP.

However, the usage of different [22–24] or rather missing [19] methods in the four studies of interest to secure pCAP diagnosis presents a notable limitation of this meta-analysis with methodological weaknesses in the detection of pCAP in all studies. Alfageme *et al*. showed a very small number of detected cases of pCAP, which might be due to the lack of using additional pneumococcal detection methods [19]. This resulted not only in a broad confidence interval for the estimation of the vaccine efficacy, but also in a potential bias of the effect estimate. However, different diagnostic measures should not influence the effect estimate (odds ratio) within the other trials [22–24]. In essence, the effect modifier “method of pneumococcal diagnostics” should be interpreted carefully, since methodological weaknesses of pneumococcal detection are not restricted to one special method alone, which would justify the exclusion of certain studies.

In summary, inconsistencies in PPV23 treatment effects to prevent pCAP could be explained on the basis of a single trial. Maruyama *et al*. [22], a trial conducted in Japan in a nursing home, the only trial using BinaxNOW^®^-assay for additional detection of pneumococci, seems to be an outlier. In essence, our meta-analysis of pCAP as endpoint which was derived from different clinical trials supports the growing evidence that PPV23 vaccination does not prevent pCAP in elderly subjects above 60 years of age. Those findings are consistent to those of other studies. The publication by Huss *et al*. i.e. also found no significant evidence that PPVs (PPV23 and others) prevent pCAP in the population of elderly or chronically ill patients (RR [95%-CI]: 1.04 [0.78–1.38]) [5]. However, it has to be taken into account that this study group consisted of different PPVs.

While the use of the PPV23 vaccination is still recommended as a standard vaccination for adults ≥60 years of age [8, 9], uncertainty remains on the effectiveness of PPV23 on pCAP and mortality in those aged 65 years and older. Recently, the Advisory Committee on Immunization Practices (ACIP) in USA recommended that all adults 65 years of age or older should receive a dose of PCV13 followed by a dose of PPV23 (at least one year later). ACIP also recommended that adults 19 years of age or older with immunocompromising conditions and/or defined underlying diseases should receive a dose of PCV13 first followed by a dose of PPV23 (at least 8 weeks later). The evidence for these recommendations was evaluated using the Grading of Recommendations, Assessment, Development, and Evaluation (GRADE) framework (http://www.cdc.gov/vaccines/vpd-vac/pneumo/vac-PCV13-adults.htm). The fact that PCV13 has been shown to be effective in preventing pCAP in the elderly [16] and the broad serotype coverage of PPV23 (23 serotypes versus 13 serotypes) were probably the main drivers for these recommendations.

This systematic review and meta-analysis supports that recommendation by ACIP in the sense that it demonstrates that–regarding all available RCTs—PPV23 vaccination (alone) does not demonstrate clear efficacy to prevent pCAP and CAP in elderly.

## Supporting Information

S1 Table Search strategy for Cochrane database. (DOCX)

S2 Table Search strategy for Embase database. (DOCX)

S3 Table Search strategy for Medline database. (DOCX)

S4 Table Operationalization of pCAP endpoint. (DOCX)

S5 Table Excluded full-text articles from Moberley *et al*. 2012. (DOCX)

S6 Table Excluded full-text articles from bibliographic literature search. (DOCX)

S7 Table PRISMA Checklist. (DOC)

## References

[1] Rozenbaum MH, Pechlivanoglou P, van der Werf TS, Lo-Ten-Foe JR, Postma MJ, Hak E. The role of Streptococcus pneumoniae in community-acquired pneumonia among adults in Europe: a meta-analysis. Eur J Clin Microbiol Infect Dis. 2013;32(3):305–16. 10.1007/s10096-012-1778-4 23242464

[2] Torres A, Blasi F, Peetermans WE, Viegi G, Welte T. The aetiology and antibiotic management of community-acquired pneumonia in adults in Europe: a literature review. Eur J Clin Microbiol Infect Dis. 2014;33(7):1065–79. 10.1007/s10096-014-2067-1 24532008 PMC4042014

[3] Welte T, Torres A, Nathwani D. Clinical and economic burden of community-acquired pneumonia among adults in Europe. Thorax. 2012;67(1):71–9. 10.1136/thx.2009.129502 20729232

[4] Cilloniz C, Polverino E, Ewig S, Aliberti S, Gabarrus A, Menendez R, et al Impact of age and comorbidity on cause and outcome in community-acquired pneumonia. Chest. 2013;144(3):999–1007. 10.1378/chest.13-0062 23670047

[5] Huss A, Scott P, Stuck AE, Trotter C, Egger M. Efficacy of pneumococcal vaccination in adults: a meta-analysis. CMAJ. 2009;180(1):48–58. 10.1503/cmaj.080734 19124790 PMC2612051

[6] Sicras-Mainar A, Ibanez-Nolla J, Cifuentes I, Guijarro P, Navarro-Artieda R, Aguilar L. Retrospective epidemiological study for the characterization of community- acquired pneumonia and pneumococcal pneumonia in adults in a well-defined area of Badalona (Barcelona, Spain). BMC Infect Dis. 2012;12:283 10.1186/1471-2334-12-283 23114195 PMC3532136

[7] van Hoek AJ, Andrews N, Waight PA, Stowe J, Gates P, George R, et al The effect of underlying clinical conditions on the risk of developing invasive pneumococcal disease in England. J Infect. 2012;65(1):17–24. 10.1016/j.jinf.2012.02.017 22394683

[8] Castiglia P. Recommendations for pneumococcal immunization outside routine childhood immunization programs in Western Europe. Adv Ther. 2014;31(10):1011–44. Epub 2014/10/11. 10.1007/s12325-014-0157-1 25300593 PMC4209094

[9] Robert Koch-Institut. Epidemiologisches Bulletin Nr. 34. Mitteilung der Ständigen Impfkommission am Robert Koch-Institut (RKI). Empfehlungen der Ständigen Impfkommission (STIKO) am Robert Koch-Institut/Stand: August 2014. 2014. Verfügbar unter: https://www.rki.de/DE/Content/Infekt/EpidBull/Archiv/2014/Ausgaben/34_14.pdf?__blob=publicationFile.

[10] European Medicines Agency (EMA). Assessment report: Prevenar 13 (EMEA/H/C/001104/II/0028). 2011.

[11] Advisory Commitee on Immunization Practices (ACIP). Summary Report—August 13, 2014. 2014. Verfügbar unter: http://www.cdc.gov/vaccines/acip/meetings/downloads/min-archive/min-2014-08.pdf.

[12] Moberley S, Holden J, Tatham DP, Andrews RM. Vaccines for preventing pneumococcal infection in adults. Cochrane Database Syst Rev. 2013;1:CD000422 Epub 02/27. 10.1002/14651858.CD000422.pub3 23440780 PMC7045867

[13] Andrews NJ, Waight PA, George RC, Slack MP, Miller E. Impact and effectiveness of 23-valent pneumococcal polysaccharide vaccine against invasive pneumococcal disease in the elderly in England and Wales. Vaccine. 2012;30(48):6802–8. 10.1016/j.vaccine.2012.09.019 23000122

[14] Salisbury CB. JCVI advice on the pneumococcal vaccination programme for people aged 65 years and older. 2011. Verfügbar unter: https://www.gov.uk/government/uploads/system/uploads/attachment_data/file/215686/dh_125244.pdf.

[15] Tomczyk S, Bennett NM, Stoecker C, Gierke R, Moore MR, Whitney CG, et al Use of 13-Valent Pneumococcal Conjugate Vaccine and 23-Valent Pneumococcal Polysaccharide Vaccine Among Adults Aged ≥65 Years: Recommendations of the Advisory Committee Immunization Practices (ACIP). Morbidity and Mortality Weekly ReportMorbidity and Mortality Weekly Report. 2014;63(37):822–5. PMC5779453 25233284

[16] Bonten MJ, Huijts SM, Bolkenbaas M, Webber C, Patterson S, Gault S, et al Polysaccharide conjugate vaccine against pneumococcal pneumonia in adults. N Engl J Med. 2015;372(12):1114–25. 10.1056/NEJMoa1408544 25785969

[17] Higgins J, Green S. Cochrane Handbook for Systematic Reviews of Interventions Version 5.1.0. 2011. Verfügbar unter: http://handbook.cochrane.org/chapter_16/16_9_2_studies_with_zero_cell_counts.htm. [Zugriffsdatum: 13.10.2015].

[18] Institute for Quality and Efficiency in Health Care (IQWIG). General Methods^a^—Version 4.2. 2015. 27403465

[19] Alfageme I, Vazquez R, Reyes N, Munoz J, Fernandez A, Hernandez M, et al Clinical efficacy of anti-pneumococcal vaccination in patients with COPD. Thorax. 2006;61(3):189–95. 16227328 10.1136/thx.2005.043323 PMC2080738

[20] Furumoto A, Ohkusa Y, Chen M, Kawakami K, Masaki H, Sueyasu Y, et al Additive effect of pneumococcal vaccine and influenza vaccine on acute exacerbation in patients with chronic lung disease. Vaccine. 2008;26(33):4284–9. 10.1016/j.vaccine.2008.05.037 18585831

[21] Kawakami K, Ohkusa Y, Kuroki R, Tanaka T, Koyama K, Harada Y, et al Effectiveness of pneumococcal polysaccharide vaccine against pneumonia and cost analysis for the elderly who receive seasonal influenza vaccine in Japan. Vaccine. 2010;28(43):7063–9. 10.1016/j.vaccine.2010.08.010 20723631

[22] Maruyama T, Taguchi O, Niederman MS, Morser J, Kobayashi H, Kobayashi T, et al Efficacy of 23-valent pneumococcal vaccine in preventing pneumonia and improving survival in nursing home residents: double blind, randomised and placebo controlled trial. BMJ. 2010;340:c1004 10.1136/bmj.c1004 20211953 PMC2834887

[23] Honkanen PO, Keistinen T, Miettinen L, Herva E, Sankilampi U, Läärä E, et al Incremental effectiveness of pneumococcal vaccine on simultaneously administered influenza vaccine in preventing pneumonia and pneumococcal pneumonia among persons aged 65 years or older. Vaccine. 1999;17:2493–500. 10418894 10.1016/s0264-410x(99)00069-9

[24] Örtqvist Å, Hedlund J, Burman L-Å, Elbel E, Höfer M, Leinonen M, et al Randomised trial of 23-valent pneumococcal capsular polysaccharide vaccine in prevention of pneumonia in middle-aged and elderly people. The Lancet. 1998;351(9100):399–403. 10.1016/s0140-6736(97)07358-3 9482293

[25] Said MA, Johnson HL, Nonyane BA, Deloria-Knoll M, O'Brien KL, Andreo F, et al Estimating the burden of pneumococcal pneumonia among adults: a systematic review and meta-analysis of diagnostic techniques. PLoS One. 2013;8(4):e60273 Epub 2013/04/09. 10.1371/journal.pone.0060273 23565216 PMC3615022

[26] Liapikou A, Polverino E, Cilloniz C, Peyrani P, Ramirez J, Menendez R, et al A worldwide perspective of nursing home-acquired pneumonia compared with community-acquired pneumonia. Respir Care. 2014;59(7):1078–85. Epub 2013/11/07. 10.4187/respcare.02788 24194575

[27] Dowell SFGRLLGLOSYY-H. Evaluation of Binax NOW, an Assay for the Detection of Pneumococcal Antigen in Urine Samples, Performed among Pediatric Patients. Clinical Infectious Diseases. 2001;32:824–5. 11229853 10.1086/319205

[28] Hamer DHE, J.; Estrella B.; MacLeod W.B.; Griffiths J.K.; Sempertegui F. Assessment of the Binax NOW Streptococcus pneumoniae Urinary Antigen Test in Children with Nasopharyngeal Pneumococcal Carriage. Clinical Infectious Diseases. 2002;34:1025–8. 11880971 10.1086/339446

[29] Horita N, Miyazawa N, Kojima R, Kimura N, Inoue M, Ishigatsubo Y, et al Sensitivity and specificity of the Streptococcus pneumoniae urinary antigen test for unconcentrated urine from adult patients with pneumonia: a meta-analysis. Respirology. 2013;18(8):1177–83. 10.1111/resp.12163 23910720

[30] Jalonen EP, J.C.; Koskela M.; Kerttula Y.; Leinonen M. Measurement of antibody responses to pneumolysin- a promising method for the presumptive aetiological diagnosis of pneumococcal pneumonia. Journal of Infection. 1989;19:127–34. 2809235 10.1016/s0163-4453(89)91864-1

[31] Leinonen M, Syrjälä H, Jalonen E, Kujala P, Herva E. Demonstration of pneumolysin antibodies in circulating immune complexes- a new diagnostic method for pneumococcal pneumonia. Serodiagn Immonuther Infect Dis. 1990;4:451–8.

[32] Scott JAGH, A.J.; Leinonen M. Validation of Immune-Complex Enzyme Immunoassays for Diagnosis of Pneumococcal Pneumonia among Adults in Kenya. Clinical and Diagnostic Laboratory Immunology. 2000:64–7. 10618279 10.1128/cdli.7.1.64-67.2000 PMC95824

[33] Musher DMM, R.; Phan H.M.; Chen G,; Baughn R.E. Nonspecificity of Assaying for IgG Antibody to Pneumolysin in Circulating Immune Complexes as a Means to Diagnose Pneumococcal Pneumonia. Clinical Infectious Diseases. 2001;32:534–8. 11181114 10.1086/318709

[34] Paton JCL, R.A.; Lee C-J.; Li J.P; Berry A.M; Mitchell T.J.; Andrew P.W.; Hansman D.; Boulnois G.J. Purification and Immunogenicity of Genetically Obtained Pneumolysin Toxoids and Their Conjugation to Streptococcus pneumoniae Type 19F Polysaccharide Infection and Immunity. 1991:2297–304. 2050399 10.1128/iai.59.7.2297-2304.1991 PMC258010

