# Supplementary material for: Efficacy of PPV23 in Preventing Pneumococcal Pneumonia in Adults at Increased Risk – A Systematic Review and Meta-Analysis
Source: PLoS One. 2016 Jan 13;11(1):e0146338. doi: 10.1371/journal.pone.0146338 (PMC4711910; doi:10.1371/journal.pone.0146338)
Supplement: S1 Table — (DOCX) [file pone.0146338.s001.docx]

S1 Table Search strategy for Cochrane database

| **Search date: 1. October 2014** |
| --- |
| **Database: EBM Reviews - Cochrane Central Register of Controlled Trials <August 2014>** |
| **Search Strategy:** |
| 1 Streptococcus pneumoniae/ (453) |
| 2 streptococcus pneumoniae.tw. (754) |
| 3 "s. pneumoniae".tw. (239) |
| 4 exp Pneumococcal Infections/ (452) |
| 5 (pneumococcal adj2 (infection* or disease*)).tw. (162) |
| 6 (pneumococc* adj5 (pneumon* or sepsis or sinusit* or meningit* or otitis media)).tw. (190) |
| 7 bacteraemic pneumon*.tw. (0) |
| 8 (invasive pneumococcal disease or ipd).tw. (115) |
| 9 or/1-8 (1370) |
| 10 exp Vaccines/ (6472) |
| 11 exp Vaccination/ (1662) |
| 12 Immunization/ (539) |
| 13 immunoprophylaxis.tw. (128) |
| 14 (immuni* or inocul* or vaccin*).tw. (12098) |
| 15 or/10-14 (12863) |
| 16 9 and 15 (469) |
| 17 Pneumococcal Vaccines/ (401) |
| 18 pneumococcal polysaccharide vaccin*.tw,nm. (157) |
| 19 ppv*.tw,nm. (522) |
| 20 pneumovax*.tw,nm. (27) |
| 21 or/16-20 (1100) |
| 22 21 and 2012:2014.(sa_year). (213) |
| 23 22 and "Humans".sa_suba. (104) |
| 24 23 and "Adult".sa_suba. (33) |
| 25 21 and 2012:2014.(sa_year). (213) |
| 26 25 and "Humans".sa_suba. (104) |
| 27 26 and "Aged".sa_suba. (30) |
| 28 24 or 27 **(50)** |
