# Supplementary material for: Efficacy of PPV23 in Preventing Pneumococcal Pneumonia in Adults at Increased Risk – A Systematic Review and Meta-Analysis
Source: PLoS One. 2016 Jan 13;11(1):e0146338. doi: 10.1371/journal.pone.0146338 (PMC4711910; doi:10.1371/journal.pone.0146338)
Supplement: S2 Table — (DOCX) [file pone.0146338.s002.docx]

S2 Table Search strategy for Embase database

| **Search date: 7. October 2014** |
| --- |
| **Database: EMBASE Classic+EMBASE <1947 to 2014 October 06>** |
| **Search Strategy:** |
| 1 Streptococcus pneumoniae/ (36941) |
| 2 streptococcus pneumoniae.tw. (21854) |
| 3 "s. pneumoniae".tw. (9026) |
| 4 exp Pneumococcal Infections/ (8665) |
| 5 (pneumococcal adj2 (infection* or disease*)).tw. (5692) |
| 6 (pneumococc* adj5 (pneumon* or sepsis or sinusit* or meningit* or otitis media)).tw. (6688) |
| 7 bacteraemic pneumon*.tw. (53) |
| 8 (invasive pneumococcal disease or ipd).tw. (3768) |
| 9 or/1-8 (51391) |
| 10 exp Vaccines/ (272392) |
| 11 exp Vaccination/ (133414) |
| 12 Immunization/ (89989) |
| 13 immunoprophylaxis.tw. (2585) |
| 14 (immuni* or inocul* or vaccin*).tw. (599103) |
| 15 or/10-14 (701587) |
| 16 9 and 15 (13548) |
| 17 Pneumococcal Vaccines/ (13052) |
| 18 pneumococcal polysaccharide vaccin*.tw,nm. (976) |
| 19 ppv*.tw,nm. (16323) |
| 20 pneumovax*.tw,nm. (1160) |
| 21 or/16-20 (35473) |
| 22 exp randomised controlled trial/ or exp single blind procedure/ or exp double blind procedure/ or exp crossover procedure/ (403415) |
| 23 (random* or placebo* or factorial* or crossover* or "cross-over" or "cross over" or volunteer* or assign* or allocat*).ab,ti. (1398392) |
| 24 ((singl* or doubl*) adj2 (blind* or mask*)).ab,ti. (171287) |
| 25 22 or 23 or 24 (1496312) |
| 26 21 and 25 (3091) |
| 27 26 and 2012:2014.(sa_year). (1000) |
| 28 limit 27 to human (942) |
| 29 middle aged.sh. or of age.tw. (3067464) |
| 30 28 and 29 **(351)** |
