# Supplementary material for: Efficacy of PPV23 in Preventing Pneumococcal Pneumonia in Adults at Increased Risk – A Systematic Review and Meta-Analysis
Source: PLoS One. 2016 Jan 13;11(1):e0146338. doi: 10.1371/journal.pone.0146338 (PMC4711910; doi:10.1371/journal.pone.0146338)
Supplement: S3 Table — (DOCX) [file pone.0146338.s003.docx]

S3 Table Search strategy for Medline database

| **Search date: 7. October 2014** |
| --- |
| **Database: Ovid MEDLINE(R) In-Process & Other Non-Indexed Citations, Ovid MEDLINE(R) Daily, Ovid MEDLINE(R) and Ovid OLDMEDLINE(R) <1946 to Present>** |
| **Search Strategy:** |
| 1 Streptococcus pneumoniae/ (18758) |
| 2 streptococcus pneumoniae.tw. (19143) |
| 3 "s. pneumoniae".tw. (7501) |
| 4 exp Pneumococcal Infections/ (16860) |
| 5 (pneumococcal adj2 (infection* or disease*)).tw. (4680) |
| 6 (pneumococc* adj5 (pneumon* or sepsis or sinusit* or meningit* or otitis media)).tw. (5372) |
| 7 bacteraemic pneumon*.tw. (48) |
| 8 (invasive pneumococcal disease or ipd).tw. (2876) |
| 9 or/1-8 (37379) |
| 10 exp Vaccines/ (176845) |
| 11 exp Vaccination/ (58589) |
| 12 Immunization/ (42889) |
| 13 immunoprophylaxis.tw. (1976) |
| 14 (immuni* or inocul* or vaccin*).tw. (483224) |
| 15 or/10-14 (538408) |
| 16 9 and 15 (9892) |
| 17 Pneumococcal Vaccines/ (4966) |
| 18 pneumococcal polysaccharide vaccin*.tw,nm. (815) |
| 19 ppv*.tw,nm. (10189) |
| 20 pneumovax*.tw,nm. (183) |
| 21 or/16-20 (20932) |
| 22 randomized controlled trial.pt. (390326) |
| 23 controlled clinical trial.pt. (89948) |
| 24 randomized.ab. (310177) |
| 25 placebo.ab. (160160) |
| 26 clinical trials as topic.sh. (173370) |
| 27 randomly.ab. (223778) |
| 28 trial.ti. (134711) |
| 29 22 or 23 or 24 or 25 or 26 or 27 or 28 (941568) |
| 30 exp animals/ not humans.sh. (4023146) |
| 31 29 not 30 (869149) |
| 32 21 and 31 (1514) |
| 33 32 and 2012:2014.(sa_year). (330) |
| 34 limit 33 to humans (280) |
| 35 middle aged.sh. or of age.tw. (4318277) |
| 36 34 and 35 **(180)** |
