# Supplementary material for: Efficacy of PPV23 in Preventing Pneumococcal Pneumonia in Adults at Increased Risk – A Systematic Review and Meta-Analysis
Source: PLoS One. 2016 Jan 13;11(1):e0146338. doi: 10.1371/journal.pone.0146338 (PMC4711910; doi:10.1371/journal.pone.0146338)
Supplement: S4 Table — (DOCX) [file pone.0146338.s004.docx]

S4 Table Operationalization of pCAP endpoint

| **Publication with pneumococcal CAP endpoints** | **Reported endpoint name** |
| --- | --- |
|  | **Operationalization** |
| Alfageme (B1) | Reported as “Pneumococcal Pneumonia” |
|  | *Presence of pneumonia (or CAP [A1]) and the isolation of S.pneumoniae from the sputum (for an adequate sample), bronchoaspirate, blood, pleural fluid, or cerebrospinal fluid. (p.190)* |
|  | Assumption: *SP* is the main pathogen in pneumonia aetiology. Therefore, the majority of pneumonia of unknown aetiology can be assumed as pneumococcal-derived. Thus, as sensitivity analysis the following endpoint will be used: |
|  | Reported as „CAP of pneumococcal or unknown aetiology“: |
|  | *A diagnosis of pneumonia (or CAP [A1]) and the isolation of S.pneumoniae from the sputum (for an adequate sample), bronchoaspirate, blood, pleural fluid, or cerebrospinal fluid or no aetiological diagnoses (unknown aetiology) (p.190)* |
| Furumoto (B2) | Not reported. |
| Kawakami (B3) | Not reported. |
| Oertqvist (B4) | Reported as “Pneumococcal pneumonia” |
|  | *Pneumonia (or CAP [A4]) and a positive culture from blood, pleural fluid, or sputum (≥10^5^ colony-forming units per mL in a purulent sample) or a positive pneumococcal antibody test. (p.400)* |
| Maruyama (B5) | Reported as “Pneumococcal pneumonia”: |
|  | *A diagnosis of pneumonia (A5) and a positive result in blood culture, pleural fluid, or sputum (10^7^ colony forming units per millilitre in a purulent sample) or a positive pneumococcal antigen test result in urine .(p.2)* |
|  | Note: This study was conducted in nursing home residents. The incidence of pneumococcal pneumonia is 20 times higher in nursing homes compared to the community. |
|  | As sensitivity analysis: Exclusion |
| Honkanen (B6) | Reported as “Pneumococcal pneumonia |
|  | *The aetiological diagnosis of pneumococcal pneumonia was based on the presence of circulating pneumolysin-specific immune complexes (PLY-IC) in one or both sera and/or a twofold rise in pneumolysin antibody level in paired serum samples.(p.2495)* |
