# Supplementary material for: Efficacy of PPV23 in Preventing Pneumococcal Pneumonia in Adults at Increased Risk – A Systematic Review and Meta-Analysis
Source: PLoS One. 2016 Jan 13;11(1):e0146338. doi: 10.1371/journal.pone.0146338 (PMC4711910; doi:10.1371/journal.pone.0146338)
Supplement: S5 Table — (DOCX) [file pone.0146338.s005.docx]

S5 Table Excluded full-text articles from Moberley 2013

| **Nr.** | **Excluded full-text articles** | **Reason for exclusion** |
| --- | --- | --- |
| 1. | Austrian R, Douglas RM, Schiffman G, Cietzee AM, Koornhof HJ, Hayden-Smith S, et al. Prevention of pneumococcal pneumonia by vaccination. *Transactions of the Association of American Physicians* 1976;89:184–94. | Wrong intervention (no PPV23) |
|  |  | No RCT |
| 2. | Austrian R. Prevention of pneumococcal infection by immunization with capsular polysaccharides of Streptococcus pneumoniae: current status of polyvalent vaccines. *Journal of Infectious Diseases* 1977;136(Suppl): 38–42. | Wrong intervention (no PPV23) |
|  |  | No RCT |
| 3. | Austrian R. Surveillance of pneumococcal infection for field trials of polyvalent pneumococcal vaccines. *National Institute of Allergy and Infectious Disease*s 1980:184–94. | Wrong intervention (no PPV23) |
|  |  | No RCT |
| 4. | Austrian R. Vaccines of pneumococcal capsular polysaccharides and the prevention of pneumococcal pneumonia. In: Beers RF Jr, Bassett EG editor(s). The Role of Immunological Factors in Infections, Allergic and Autoimmune Processes. New York: *Raven Press*, 1976: Chapter 8, 79-89. | Wrong intervention (no PPV23) |
|  |  | No RCT |
| 5. | Davis AL, Aranda CP, Schiffman G, Christianson LC. Pneumococcal infection and immunologic response to pneumococcal vaccine in chronic obstructive pulmonary disease. A pilot study. *Chest* 1987;92:204–12. | Wrong intervention |
|  |  | (PPV14) |
| 6. | Gaillat J, Zmirou D, Mallaret MR, Rouhan D, Bru JP, Stahl JP, et al.Clinical trial of an anti-pneumococcal vaccine in elderly people living in institutions. *Revue d’Epidemiologie et de Sante Publique* 1985;33:437–44. | Wrong intervention (PPV14) |
| 7. | Kaufman P. Pneumonia in old age. *Archives of Internal Medicine* 1947;79:518–31. | Wrong intervention (PPV2, PPV3) |
|  |  | No RCT |
| 8. | Klastersky J, Mommen P, Cantraine F, Safary A. Placebo controlled pneumococcal immunization in patients with bronchogenic carcinoma. *European Journal of Cancer and Clinical Oncology* 1986;22:807–13. | Wrong intervention (PPV17) |
| 9. | Klugman KP, Hayden Smith SW, Koornhof HJ. Evidence that prevention of carriage by pneumococcal capsular vaccines may be the mechanism of protection from pneumococcal pneumonia. *South African Journal of Epidemiology and Infection* 2011;26(4):221–4. | Wrong intervention (PPV6, PPV13) |
|  |  | Wrong population (newly recruited miners) |
| 10. | Koivula I, Sten M, Leinonen M, Makela PH. Clinical efficacy of pneumococcal vaccine in the elderly: a randomized, single-blind population-based trial. *American Journal of Medicine* 1997;103:281–90. | Wrong intervention (PPV14) |
| 11. | Leech JA, Gervais A, Ruben FL. Efficacy of pneumococcal vaccine in severe chronic obstructive pulmonary disease. *Canadian Medical Association Journal* 1987;136:361–5. | Wrong intervention (PPV14) |
| 12. | Riley ID, Andrews M, Howard R, Tarr PI, Pfeiffer M, Challands P, et al.Immunization with polyvalent pneumococcal vaccine. Reduction of adult respiratory mortality in a New Guinea Highlands community. *Lancet* 1977;1(8026):1338–41. | Wrong intervention (PPV14) |
| 13. | Simberkoff M, Cross A, Al-Ibrahim M, Baltch A, Geiseler P, Nadler J. Efficacy of pneumococcal vaccine in highrisk patients. *New England Journal of Medicine* 1986;315: 1318–27. | Wrong intervention (PPV14) |
| 14. | Smit P, Oberholzer D, Hayden-Smith S, Koornhof H, Hilleman M. Protective efficacy of pneumococcal polysaccharide vaccines. *JAMA* 1977;238:2613–6. | Wrong population (mine workers without increased danger) |
|  |  | Wrong intervention (PPV6 and PPV12) |
