# Supplementary material for: Efficacy of PPV23 in Preventing Pneumococcal Pneumonia in Adults at Increased Risk – A Systematic Review and Meta-Analysis
Source: PLoS One. 2016 Jan 13;11(1):e0146338. doi: 10.1371/journal.pone.0146338 (PMC4711910; doi:10.1371/journal.pone.0146338)
Supplement: S6 Table — (DOCX) [file pone.0146338.s006.docx]

S6 Table Excluded full-text articles from bibliographic literature search

| **Nr.** | **Excluded full-text articles** | **Reason for exclusion** |
| --- | --- | --- |
| 1. | E. A. Clutterbuck, R. Lazarus, L. M. Yu, J. Bowman, E. A. Bateman, L. Diggle, B. Angus, T. E. Peto, P. C. Beverley, D. Mant and A. J. Pollard (2012). Pneumococcal conjugate and plain polysaccharide vaccines have divergent effects on antigen-specific B cells. *Journal of infectious diseases* 205 (9), 1408-16 | Wrong endpoints (B-cell responses) |
|  |  |  |
| 2. | M. T. Dransfield, S. Harnden, R. L. Burton, R. K. Albert, W. C. Bailey, R. Casaburi, J. Connett, J. A. Cooper, G. J. Criner, J. L. Curtis, M. K. Han, B. Make, N. Marchetti, F. J. Martinez, C. McEvoy, M. H. Nahm, D. E. Niewoehner, J. Porszasz, J. Reilly, P. D. Scanlon, S. M. Scharf, F. C. Sciurba, G. R. Washko, P. G. Woodruff, S. C. Lazarus and N. C. C. R. Network (2012). Long-term comparative immunogenicity of protein conjugate and free polysaccharide pneumococcal vaccines in chronic obstructive pulmonary disease. Clinical infectious diseases : an official publication of the *Infectious Diseases Society of America* 55 (5), e35-44 | Wrong control group (PCV7) |
|  |  |  |
| 3. | C. Forstner, S. Plefka, S. Tobudic, H. M. Winkler, K. Burgmann and H. Burgmann (2012). Effectiveness and immunogenicity of pneumococcal vaccination in splenectomized and functionally asplenic patients. *Vaccine* 30 (37), 5449-52 | No RCT |
|  |  |  |
| 4. | Y.-L. Ho, A. P. Brandao, M. C. de Cunto Brandileone and M. H. Lopes (2013). Immunogenicity and safety of pneumococcal conjugate polysaccharide and free polysaccharide vaccines alone or combined in HIV-infected adults in Brazil. *Vaccine* 31 (37), 4047-53 | Wrong population (HIV-infected patients) |
|  |  |  |
| 5. | L. A. Jackson, A. Gurtman, K. Rice, K. Pauksens, R. N. Greenberg, T. R. Jones, D. A. Scott, E. A. Emini, W. C. Gruber and B. Schmoele-Thoma (2013). Immunogenicity and safety of a 13-valent pneumococcal conjugate vaccine in adults 70 years of age and older previously vaccinated with 23-valent pneumococcal polysaccharide vaccine. *Vaccine* 31 (35), 3585-3593 | Wrong control group (PCV13) |
|  |  |  |
| 6. | L. A. Jackson, A. Gurtman, M. van Cleeff, K. U. Jansen, D. Jayawardene, C. Devlin, D. A. Scott, E. A. Emini, W. C. Gruber and B. Schmoele-Thoma (2013). Immunogenicity and safety of a 13-valent pneumococcal conjugate vaccine compared to a 23-valent pneumococcal polysaccharide vaccine in pneumococcal vaccine-naive adults. *Vaccine* 31 (35), 3577-3584 | Wrong control group (PCV13) |
|  |  |  |
| 7. | L. A. Jackson, A. Gurtman, M. van Cleeff, R. W. Frenck, J. Treanor, K. U. Jansen, D. A. Scott, E. A. Emini, W. C. Gruber and B. Schmoele-Thoma (2013). Influence of initial vaccination with 13-valent pneumococcal conjugate vaccine or 23-valent pneumococcal polysaccharide vaccine on anti-pneumococcal responses following subsequent pneumococcal vaccination in adults 50 years and older. *Vaccine* 31 (35), 3594-602 | Wrong control group (PCV13) |
|  |  | Wrong endpoints (opsonophagocytic activity, local and systemic reactions) |
| 8. | C. Juergens, P. J. T. De Villiers, K. Moodley, D. Jayawardene, K. U. Jansen, D. A. Scott, E. A. Emini, W. C. Gruber and B. Schmoele-Thoma (2014). Safety and immunogenicity of 13-valent pneumococcal conjugate vaccine formulations with and without aluminum phosphate and comparison of the formulation of choice with 23-valent pneumococcal polysaccharide vaccine in elderly adults: A randomized open-label trial. *Human Vaccines and Immunotherapeutics* 10(5), 1343-1353 | Wrong control group (PCV13) |
|  |  |  |
| 9. | C. R. MacIntyre, I. Ridda, Z. Gao, A. M. Moa, P. B. McIntyre, J. S. Sullivan, T. R. Jones, A. Hayen and R. I. Lindley (2014). A randomized clinical trial of the immunogenicity of 7-valent pneumococcal conjugate vaccine compared to 23-valent polysaccharide vaccine in frail, hospitalized elderly. *PLoS ONE* 9 (4), Date: 23 Apr | Wrong control group (PCV7) |
|  |  | Wrong endpoints (opsonophagocytic activity) |
| 10. | K. Pauksens, A. C. Nilsson, M. Caubet, T. G. Pascal, P. Van Belle, J. T. Poolman, P. G. Vandepapeliere, V. Verlant and P. E. Vink (2014). Randomized controlled study of the safety and immunogenicity of pneumococcal vaccine formulations containing PhtD and detoxified pneumolysin with alum or adjuvant system AS02Vin elderly adults. *Clinical and Vaccine Immunology* 21 (5), 651-660 | Wrong control group (protein vaccines) |
|  |  |  |
| 11. | E. P. Schlaudecker, M. C. Steinhoff, S. B. Omer, E. Roy, S. E. Arifeen, C. N. Dodd, M. Altaye, R. Raqib, R. F. Breiman and K. Zaman (2012). Antibody persistence in mothers one year after pneumococcal immunization in pregnancy. *Vaccine* 30 (34), 5063-6 | Wrong control group (influenza vaccination) |
|  |  | Wrong endpoints (immune responses) |
|  |  | Wrong population (pregnant women, 18-36 years of age) |
| 12. | K. L. Slayter, J. Singer, T. C. K. Lee, H. Kayhty and W. F. Schlech (2013). Immunization against pneumococcal disease in HIV-infected patients: Conjugate versus polysaccharide vaccine before or after reconstitution of the immune system (CTN-147). *International Journal of STD and AIDS* 24 (3), 227-231 | Wrong population (HIV-infected patients) |
|  |  | Wrong control group (PCV7) |
| 13. | S. Tobudic, V. Plunger, G. Sunder-Plassmann, M. Riegersperger and H. Burgmann (2012). Randomized, single blind, controlled trial to evaluate the prime-boost strategy for pneumococcal vaccination in renal transplant recipients. *PLoS ONE* [Electronic Resource] 7(9), e46133 | Wrong control group (PCV7) |
|  |  | Wrong endpoints (immunogenicity endpoints) |
| 14. | C. Sadlier, N. Conlon, C. Rock, A. Brown, S. O'Dea, J. Dunne and C. Bergin (2014). Immunological efficacy of a prime-boost vaccine strategy combining the 13-valent pneumococcal conjugate vaccine (PCV13) followed by the 23-valent pneumococcal polysaccharide vaccine (PPV23) versus PPV 23 alone in HIV-infected adults. *HIV Medicine* 15, 5-6 | Wrong population (HIV-infected patients) |
|  |  | Wrong endpoints (opsonophagocytic activity) |
|  |  | Wrong publication type (conference paper) |
